# Sleep disturbances and semen quality in an Italian cross sectional study

**DOI:** 10.1186/s12610-017-0060-0

**Published:** 2017-08-21

**Authors:** Paola Viganò, Francesca Chiaffarino, Viviana Bonzi, Andrea Salonia, Elena Ricci, Enrico Papaleo, Paola Agnese Mauri, Fabio Parazzini

**Affiliations:** 1 Reproductive Sciences Laboratory, Division of Genetics and Cell Biology, IRCCS Ospedale San Raffaele, Milan, Italy; 2 Dipartimento della Donna, del Neonato e del Bambino, Fondazione IRCCS Ca’ Granda Ospedale Maggiore Policlinico, Via Commenda 12, 20122 Milano, Italy; 3 Obstetrics and Gynecology Unit, IRCCS San Raffaele Scientific Institute, Milan, Italy; 4 Division of Experimental Oncology/Unit of Urology; URI; IRCCS Ospedale San Raffaele, Milan, Italy; 5 Dipartimento di Scienze Cliniche e di Comunità, Università degli studi di Milano, Milano, Italy

**Keywords:** Sperm parameters, Sleep quality, Sleep parameters, Sperm motility, Paramètres spermatiques, Qualité du sommeil, Paramètres du sommeil, Mobilité des spermatozoïdes

## Abstract

**Introduction:**

In order to obtain information about the relationship between sleep disturbances and sperm parameters, we analyzed data from a study conducted in a Italian Fertility Clinic, in men of couples seeking help for infertility.

**Patients and methods:**

Male partners with or without a medical history of reproductive organ diseases (cryptorchidism, varicocele, orchitis, testicular torsion) were eligible for the study. There were 382 men evaluated from May 2014 to November 2016, all of whom completed a self-administered questionnaire on general lifestyle habits. Then all men underwent semen analysis. A total of 382 men aged 26 to 67 years (median age 39 year interquartile range 37–42) were recruited.

**Main results:**

A total of 46.3% reported having sleep disturbances. In multivariate analysis, in absence of reproductive organ diseases, semen volume was lower in patients with difficulty in initiating sleep (2.0 ml, IQR 1.5–3.0 vs 3.0 ml, IQR 2.0–3.3, *p* = .01), whereas in presence of reproductive organ diseases motility A was lower in patients with early morning awakening (25.0%, IQR 15.0–35.0 vs. 40.0%, IQR 30.0–50.0, *p* = .001). In overweight men, semen volume was lower in patients with difficulty in initiating sleep (2.0 ml, IQR 1.5–3.0 vs 3.0 ml, IQR 2.0–3.0, *p* = .03). Moreover, among current smokers, patients with difficulty in initiating sleep had semen volume lower (1.5 ml, IQR 1.5–2.5 vs 3.0 ml, IQR 2.0–3.5, *p* = .0003) and sperm concentration higher (40 millions/ml, IQR 15–60 vs 10 millions/ml, IQR 5–50 *p* = .03) but total sperm count was not significant different.

**Conclusion:**

Further studies are necessary to elucidate the relationship between sleep quality and semen parameters, which may have important public health implication.

**Electronic supplementary material:**

The online version of this article (doi:10.1186/s12610-017-0060-0) contains supplementary material, which is available to authorized users.

## Résumé

**Contexte:**

Dans le but d’obtenir des informations concernant les relations entre troubles du sommeil et paramètres spermatiques, nous avons analysé les données recueillies au cours d’une étude menée dans une Clinique de la Fertilité, en Italie, chez les hommes de couples en recherche d’aide pour infécondité.

**Patients et Méthodes:**

Etaient éligibles pour l’étude tous les partenaires masculins avec ou sans antécédents médicaux d’affections des organes reproducteurs (mal descente testiculaire, varicocèle, torsion du cordon spermatique). De mai 2014 à novembre 2016, 382 hommes ont été évalués, tous ayant rempli un questionnaire auto-administré sur les habitudes générales du mode de vie. Puis chacun a eu une analyse de sperme. Au total, 382 hommes âgés de 26 à 67 ans (médiane: 39 ans; intervalle interquartile (IQR): 37–42) ont été recrutés.

**Résultats:**

Au total, 46% des hommes ont rapporté avoir des troubles du sommeil. En analyse multivariée, en l’absence d’affections des organes reproducteurs, le volume de sperme était abaissé chez les patients qui présentaient des difficultés d’endormissement (2.0 mL, IQR 1.5–3.0 vs 3.0 mL, IQR 2.0–3.3, *p* = .01); alors qu’en présence d’affections des organes reproducteurs la mobilité A était plus basse chez ceux qui avaient un réveil matinal précoce (25.0%, IQR 15.0–35.0 vs. 40.0%, IQR 30.0–50.0, *p* = .001). Parmi les hommes en surpoids, le volume de sperme était plus faible chez ceux qui présentaient des difficultés d’endormissement (2.0 mL, IQR 1.5–3.0 vs 3.0 mL, IQR 2.0–3.0, *p* = .03). De plus, parmi les fumeurs, ceux qui avaient des difficultés d’endormissement présentaient un volume de sperme abaissé (1.5 mL, IQR 1.5–2.5 vs 3.0 mL, IQR 2.0–3.5, *p* = .0003) et une concentration de spermatozoïdes plus grande (40 millions/mL, IQR 15–60 vs 10 millions/mL, IQR 5–50 *p* = .03); mais la quantité totale de spermatozoïdes dans l’éjaculat n’était pas significativement différente.

**Conclusion:**

Des études supplémentaires sont nécessaires pour déterminer la nature des relations entre qualité du sommeil et paramètres spermatiques, ce qui pourrait avoir d’importantes implications sur la santé publique.

## Introduction

Sleep disturbances cause higher risk of developing several pathological condition, as hypertension, diabetes and gastrointestinal disorders [1–3].

It has been reported a decrease in semen quality in general men population and several research were carried out on this topic [4] but very few studies have examined the relationship between sleep disorder and semen quality. In a cross-sectional study among 953 young Danish men from the general population, an inverse U-shaped association between self-reported sleep disturbances and semen quality was found [5]. Likewise, a recent study found an inverse U-shaped association between sleep duration and two semen parameters (semen volume and total sperm number) [6].

We analyzed data from a study on the relationship between sleep disturbances and sperm parameters in men of couples seeking help for infertility.

## Patients and methods

Couples with primary infertility, seeking evaluation and treatment at the Fertility Clinic of San Raffaele Scientific Institute in Milan were invited to participate in a cross-sectional study on the relationship between lifestyle patterns and fertility. Male partners who agreed to participate, consecutively observed during the study period, with or without a medical history of reproductive organ diseases (cryptorchidism, varicocele, orchitis, testicular torsion) were eligible for the present analysis. Exclusion criteria encompassed of the presence of systematic and chronic diseases (e.g. renal and liver disease, type 2 diabetes), osteometabolic disorders and malignancies. To reduce geographic and racial heterogeneity, only Caucasian men were enrolled. Based on these data, 18 men have been excluded. There were 382 men evaluated from May 2014 to November 2016, all of whom completed an assessment questionnaire. All procedures were in accord with the Helsinki Declaration and all participants provided written informed consent. The study protocol was approved by the Ethical Review Board of San Raffaele Scientific Institute, Milano, Italy.

Each man was asked to complete a self-administered standard questionnaire on sociodemographic characteristic and general lifestyle habits (see Additional file 1). Moreover, men were asked to fill a standard sheet to document common use of drugs or presence of chronic diseases. Reproductive organ diseases were self-reported and then checked with medical record.

In order to evaluate sleep disturbances, the participants were also asked to fulfill some questions regarding subjective insufficient sleep, difficulty in initiating sleep, difficulty in maintaining sleep and early morning awakening changing in part those proposed by Ohida et al. [7].

Men were instructed to abstain from intercourse for 3 to 5 days before semen analysis. Only complete samples obtained by masturbation and collected into a plastic container were evaluated after labelling them with the date and time of collection. All seminal fluid examinations were carried out by the same biologist (VB). The semen sample was immediately delivered to the laboratory after collection in the clinic and incubated in a 37 °C incubator. Duration of complete liquefaction (<1 h) was documented, until 1 h was reached. Semen analysis was performed with standardized methods according to the newest World Health Organization semen analysis manual. Semen volume was measured by weighing, assuming a semen density of 1.0 g/ml; sperm concentration was assessed in duplicate using an Improved Neubauer haemocytometer, with volumes of semen being dispensed using a Gilson Microman M25, M50 or M250 positive displacement pipette (Gilson UK, Luton, UK) as appropriate to the dilution being made. The following variables were taken into consideration: volume (mL), sperm concentration (n/mL) and motility (%). Sperm motility was graded into total (progressive + non-progressive motility) and progressive motility. Total sperm count (volume × sperm concentration) was also calculated. Reference values from the WHO semen analysis manual were used to assess sperm concentration and motility [8]. In presence of a pathological semen analysis, men were also referred to an andrological examination. The SEMinal QUAlity studies (SEMQUA) checklist was followed to improve accuracy and transparency of the study [9]. Both an internal and external quality control programme [European Society of Human Reproduction and Embryology (ESHRE)] has been established in the laboratory in order to control random and systematic errors and interlaboratory differences. All the personnel was trained based on the ESHRE Special Interest Group in Andrology Basic Semen Analysis Course.

Because of the non-normal (skewed) distributions of semen quality, at univariate analysis, semen parameters were described as medians and interquartile range (IQR) and groups were compared by means of Mann-Withney U test. To perform a multivariate analysis including potential confounders, semen parameters were cubic-root transformed and compared using a general linear model on transformed values. All reported *P*-values are based on two-sided tests and compared to a significance level of 5%.

## Results

A total of 382 men aged 26 to 67 years (median age 39 year interquartile range 37–42) were recruited and answered to questionnaire. As shown in Table 1, at univariate analysis, among current smokers, smoking 10 or more cigarettes per day was associated with lower semen volume, as compared to smoking 9 or less cigarettes (*p* = .05). The presence of reproductive organ diseases was associated with lower concentration (*p* = .002), total sperm count (*p* = .04) and motility A + B (*p* = .01). A + B motility was also lower in overweight men (Body Mass Index-BMI ≥ 25.0; *p* = .05). A total of 46.3% reported having insufficient sleep. Among these, 37.8% had difficulty in initiating sleep, 24% lying awake most of the night and 46.2% had early morning awakening. Lying awake most of the night was associated with lower A + B or A motility (respectively *p* = .02 and *p* = .04) whereas men with difficulty in initiating sleep had lower semen volume (*p* = .003) and higher sperm concentration (*p* = .05).

**Table 1:** Median semen parameters (interquartile range) according to selected characteristics and to sleep disturbances

	N patients	Semen volume (ml)	Sperm concentration (millions/ml)	Total sperm count (millions)	Motility (A + B) (%)	Motility (A) (%)
Overall	382	2.5 (2.0–3.0)	30.0 (10.0–50.0)	60.0 (23.8–120.0)	50.0 (40.0–60.0)	35.0 (20.0–45.0)
Age (years)						
≤ 37	125	2.5 (2.0–3.0)	20.0 (7.0–50.0)	60.0 (20.0–120.0)	45.0 (35.0–55.0)	30.0 (20.0–40.0)
38–41	114	2.5 (2.0–3.0)	30.0 (15.0–55.0)	70.0 (30.0–120.0)	50.0 (40.0–60.0)	35.0 (25.0–45.0)
≥ 42	111	2.5 (2.0–3.0)	30.0 (10.0–50.0)	60.0 (22.5–125.0)	50.0 (30.0–60.0)	35.0 (20.0–50.0)
Reproductive organ diseases						
No	291	2.5 (2.0–3.0)	**30.0 (15.0–50.0)** ^**a**^	**60.0 (30.0–120.0)** ^**b**^	**50.0 (40.0–60.0)** ^**c**^	35.0 (25.0–45.0)
Yes	91	2.5 (2.0–3.5)	**20.0 (5.0–40.0)**	**50.0 (12.5–120.0)**	**40.0 (30.0–50.0)**	30.0 (20.0–40.0)
Children						
No	310	2.5 (2.0–3.0)	30.0 (10.0–50.0)	60.0 (21.0–120.0)	50.0 (40.0–55.0)	35.0 (20.0–45.0)
Yes	72	2.5 (2.0–3.0)	30.0 (10.0–50.0)	62.5 (30.0–120.0)	50.0 (37.5–60.0)	40.0 (25.0–50.0)
BMI						
< 25.00	195	2.5 (2.0–3.0)	30.0 (15.0–50.0)	61.3 (30.0–120.0)	**50.0 (40.0–60.0)** ^**d**^	35.0 (25.0–45.0)
≥ 25.00	185	2.5 (2.0–3.0)	30.0 (10.0–50.0)	60.0 (20.0–112.5)	**45.0 (30.0–55.0)**	30.0 (20.0–45.0)
Smoking						
No	278	2.5 (2.0–3.0)	25.0 (10.0–50.0)	60.0 (22.5–125.0)	50.0 (35.0–60.0)	35.0 (25.0–45.0)
Yes	104	2.5 (2.0–3.0)	30.0 (10.0–50.0)	60.0 (27.5–100.0)	45.0 (40.0–60.0)	35.0 (20.0–45.0)
0–9 cig/day	47	**2.5 (2.0–3.0)** ^**e**^	25.0 (10.0–40.0)	51.3 (20.0–90.0)	45.0 (30.0–50.0)	35.0 (20.0–45.0)
≥ 10 cig/day	57	**2.0 (1.5–3.0)**	30.0 (15.0–60.0)	61.3 (30.0–100.0)	50.0 (40.0–60.0)	37.5 (30.0–45.0)
Alcohol intake						
Never	184	2.5 (2.0–3.0)	20.0 (10.0–50.0)	52.5 (15.0–100.0)	50.0 (40.0–60.0)	35.0 (25.0–45.0)
Ever	198	2.5 (2.0–3.0)	30.0 (10.0–50.0)	75.0 (25.0–120.0)	50.0 (35.0–60.0)	35.0 (20.0–45.0)
Subjective insufficient sleep						
Yes	177	2.5 (1.5–3.0)	30.0 (10.0–50.0)	60.0 (20.0–120.0)	45.0 (40.0–60.0)	35.0 (20.0–45.0)
No	205	2.5 (2.0–3.0)	25.0 (10.0–50.0)	62.5 (25.0–120.0)	50.0 (35.0–60.0)	35.0 (25.0–45.0)
If answered Yes:						
Difficulty in initiating sleep						
Yes	67	**2.0 (1.5–3.0)** ^**f**^	**30.0 (10.0–60.0)** ^**g**^	60.0 (30.0–120.0)	50.0 (40.0–60.0)	40.0 (25.0–45.0)
No	107	**2.8 (2.0–3.5)**	**25.0 (8.0–50.0)**	47.5 (17.0–137.5)	45.0 (35.0–55.0)	30.0 (20.0–45.0)
Lying awake most of the night						
Yes	41	2.8 (1.5–3.0)	20.0 (10.0–40.0)	42.5 (15.0–120.0)	**40.0 (35.0–50.0)** ^**h**^	**30.0 (20.0–35.0)** ^**i**^
No	130	2.5 (2.0–3.0)	30.0 (10.0–60.0)	60.0 (22.5–120.0)	**50.0 (40.0–60.0)**	**40.0 (20.0–50.0)**
Early morning awakening						
Yes	80	2.5 (2.0–3.0)	30.0 (9.0–50.0)	60.0 (23.0–130.0)	45.0 (35.0–60.0)	35.0 (20.0–45.0)
No	93	2.3 (1.5–3.0)	30.0 (10.0–50.0)	51.3 (20.0–120.0)	45.0 (40.0–55.0)	35.0 (20.0–45.0)

Bold results are statistically significant

*BMI* body mass index

^a^p = .002

^b^p = .04

^c^p = .01

^d^p = .03

^e^p = .05

^f^p = .003

^g^p = .05

^h^p = .01

^i^p = .04

We performed the multivariate analysis of sleep disturbances in strata of reproductive organ diseases, BMI and smoking, characteristics significantly associated with at least one semen variable, as shown in Table 1. In absence of reproductive organ diseases, semen volume was lower in patients with difficulty in initiating sleep (2.0 ml, IQR 1.5–3.0 vs 3.0 ml, IQR 2.0–3.3, *p* = .01), whereas in presence of reproductive organ diseases motility A was lower in patients with early morning awakening (25.0%, IQR 15.0–35.0 vs. 40.0%, IQR 30.0–50.0, *p* = .001). In overweight men, semen volume was lower in patients with difficulty in initiating sleep (2.0 ml, IQR 1.5–3.0 vs 3.0 ml, IQR 2.0–3.0, *p* = .03). Moreover, among current smokers, patients with difficulty in initiating sleep had semen volume lower (1.5 ml, IQR 1.5–2.5 vs 3.0 ml, IQR 2.0–3.5, *p* = .0003) and sperm concentration higher (40 millions/ml, IQR 15–60 vs 10 millions/ml, IQR 5–50 *p* = .03) but total sperm count was not significant different.

## Discussion

In our study some of sleep disturbances appeared associated to semen quality; in particular lying awake most of the night were associated to lower motility and difficulty in initiating sleep appeared associated to lower semen volume and higher sperm concentration. In absence of reproductive organ diseases, semen volume was lower in patients with difficulty in initiating sleep, whereas in presence of diseases motility A was lower in patients with early morning awakening. Moreover, in overweight men, semen volume was lower in patients with difficulty in initiating sleep. In current smokers, patients with difficulty in initiating sleep had semen volume lower and sperm concentration higher, but no significant difference of total sperm count.

Potential limitations of this study should be considered.

The information about sleep disturbances was self-reported, thus some misclassification may occur. Moreover, we analyzed men of couples attending to a Fertility Clinic and as such the results of this study could not be generalized. Other sources of bias, including selection or confounding factors, are unlikely to have produced marked effects, especially considering that men were interviewed in the same institution, before semen analysis results were available, and that participation was practically complete. Lastly, a main limitation of this study is that information on sperm morphology was not collected.

Among the strengths of this study, we had the opportunity to analyze the role of sleep disturbances in men with or without other condition associated with infertility and also of taking into account the role of potential biases, such as smoking and BMI, that have been reported to be associated with semen quality [10], but residual confounding may still have been present.

Few data are available on the relationship between sleep disturbances and semen quality. A Danish cross-sectional study found an association between sleep disturbances and sperm concentration, total sperm count and morphologically normal spermatozoa but lack to find an association of sleep with motility [5]. We found, conversely, some characteristics of poor sleep quality associated with lower motility, lower semen volume and higher sperm concentration. In a cohort of Chinese male students, either excessive or restricted sleep duration was associated with decreased semen volume and total sperm number [6].

About the biological mechanism underlying the relationship between sleep disturbances and semen quality, it has been observed that a lack of sleep increased cortisol level, which is able to cause sleep interruptions decreasing thus the duration in hours and sleep quality and testosterone level [11, 12]. Recent evidence has shown that sleep deprivation in the animal results in structural testicular abnormalities and reduced sperm quality (in particular sperm motility reduction) [13]. Moreover, Jensen et al. found no associations between serum reproductive hormone levels and sleeping disturbances [5].

In addition sleep disturbances can be associated with an unhealthier lifestyle: e.g. smoking and overweight, factors influencing semen characteristics.

Because sleep disturbances and a decrease in semen quality are increasing in contemporary society, further studies in order to elucidate the relationship between sleep quality and semen parameters may have important public health implication.

## Additional file

Additional file 1: Questionnaire RLS_Sleep. (PDF 376 kb)

## Abbreviations

BMI: Body Mass Index
ESHRE: European Society of Human Reproduction and Embryology
IQR: interquartile range
SEMQUA: SEMinal QUAlity studies
