# Supplementary material for: Sleep disturbances and semen quality in an Italian cross sectional study
Source: Basic Clin Androl. 2017 Aug 21;27:16. doi: 10.1186/s12610-017-0060-0 (PMC5563907; doi:10.1186/s12610-017-0060-0)
Supplement: Additional file 1: — Questionnaire RLS_Sleep. (PDF 376 kb) [file 12610_2017_60_MOESM1_ESM.pdf]

Scheda studio RLS e disturbi del sonno infertilità

Studio
N° paziente

		g m a
Data intervista		/_/_/ /_/_/_/_/

Età

Stato civile:	mai sposato/a	=1	coniugato/a	=2
	separato/a, divorziato/a	=3	vedovo/a	=4
			convivente	=5

Titolo di studio, paziente ________________________ anni di istruzione /_/_/

Titolo di studio, partner ________________________ anni di istruzione /_/_/

Professione paziente ____ /_/_/

Professione partner ____ /_/_/

Numero totale gravidanze /__/

Fumo

Non fumatore=1; fumatore=2

se fumatore: sigarette, n°/die

COME DESCRIVEREBBE IL SUO LIVELLO DI ATTIVITA' FISICA ?

LAVORO/CASA

1 = MOLTO FATICOSA (muratore, atleta e ballerino professionisti)
2 = FATICOSA (agricoltore, operaio industria metalmeccanica, ecc.)
3 = MEDIA (rappresentante, cameriere, cuoco, maestra d'asilo, casalinga con lavori pesanti o bambini piccoli)
4 = IN PIEDI (commessa, insegnante, laboratorista, casalinga, lavori leggeri)

5 = ESCLUSIVAMENTE O PRINCIPALMENTE SEDUTA (impiegato d'ufficio, meccanico di precisione, studente) /__/

SPORT TEMPO LIBERO, SPOSTAMENTI IN BICICLETTA

1 = > 7 ore alla settimana di attività fisica
2 = 5-7 ore alla settimana di attività fisica
3 = 2-4 ore alla settimana di attività fisica
4 = < 2 ore alla settimana di attività fisica /__/

Peso

Peso (in Kg) /_/_/_/

Altezza (in cm) /_/_/_/

Consuma alcolici?
/1/ No /2/ Si

Se Sì indicare consumo medio in bicchieri die /__/__/

Studio

CARD 2
Anamnesi patologica

N° paziente
caso/controllo
N° card

Ha avuto le seguenti patologie?

Diabete mellito ....

Ipertensione (trattata con farmaci) ....

Varicocele..

Orchite ....

Patologie della tiroide (specificare) ....

Interventi per torsione
testicolare..

Altro ....

QUESTIONARIO QUALITA' DEL SONNO

Le capita mai di avere disturbi del sonno? Si no

Con che frequenza? 1/mese 1/sett 3-4/ sett. Tutte le sere

Il suo problema riguarda una difficoltà:

-**iniziale** ad addormentarsi, oltre 30-40 min? Si no

-**centrale**? Veglia prolungata intrasonno si no

Uno o più/numerosi risvegli notturni uno più

Difficoltà (D) o impossibilità (I) a riaddormentarsi D I

-**terminale**? Risveglio mattutino precoce si no

Associato a questo disturbo le capita anche di avere?

- disturbi del respiro, esempio di tipo ostruttivo, russamento? Si no

QUESTIONARIO SINDROME GAMBE SENZA RIPOSO

1)LE CAPITA DI AVERE UNA SENSAZIONE SPIACEVOLE ALLE GAMBE ASSOCIATA AD UNA URGENZA O BISOGNO DI MUOVERE LE GAMBE? SI NO

1)QUESTA SENSAZIONE/SINTOMO LE CAPITA PRINCIPALMENTE O SOLO A RIPOSO E MIGLIORA CON IL MOVIMENTO ? SI NO

1)QUESTE SENSAZIONI/SINTOMI SONO PEGGIORI DI SERA O DI NOTTE PIUTTOSTO CHE DI GIORNO ?
SI NO

1)CON CHE FREQUENZA LE CAPITANO QUESTE SENSAZIONI ?

MENO DI UNA VOLTA ALL'ANNO SI NO

PIU' DI UNA VOLTA ALL'ANNO MA MENO DI UNA VOLTA AL MESE SI NO

UNA VOLTA OGNI MESE SI NO

2-4 VOLTE AL MESE SI NO

2-3 VOLTE ALLA SETTIMANA SI NO

4-5 VOLTE ALLA SETTIMANA SI NO

6-7 VOLTE ALLA SETTIMANA SI NO
